# Fertility awareness, attitudes towards parenting, and knowledge about Assisted Reproductive Technology among university students in Argentina

**DOI:** 10.5935/1518-0557.20210019

**Published:** 2021

**Authors:** Ludmila Jurkowski, Rocio Manubens, Julieta Olivera Ryberg, Mariela Rossi

**Affiliations:** 1 Psychology Department, Universidad de San Martin, Buenos Aires, Argentina; 2 Psychology Research Department, Universidad de Belgrano, CABA, Argentina; 3 Psychology Research Department, Universidad de la Marina Mercante, CABA, Argentina; 4 Psychology Department, Fertilis Medicina Reproductiva, Buenos Aires, Argentina

**Keywords:** fertility awareness, parenting attitudes, assisted reproductive technology, university students

## Abstract

**Objective:**

The aim of this study was to assess the fertility awareness, attitudes towards parenting, and knowledge about Assisted Reproductive Technology of Argentinian university students.

**Methods:**

This naturalistic, cross-sectional and quantitative study included the translation into Spanish of the Swedish Fertility Awareness Questionnaire; adjustments were also made to fit the questionnaire to local cultural norm. Through a snowball design using social media, university students were contacted in June 2019 and asked to complete an anonymous online self-report survey.

**Results:**

A total of 680 students (83.2% females and 16.4% males) aged 24.7 years on average (SD=5.6) answered the questionnaire. Approximately 70% believed they had high levels of knowledge about human reproduction; nevertheless, 46% thought that women could get pregnant in any stage of the cycle; 36.2% believed that fertility in women decreased between the ages of 45 and 50, 33.2% between the ages of 40 and 45, and 25.9% between the ages of 35 and 40 years. Half of the studied population thought that the chances of getting pregnant during the ovulation period ranged between 80-100%. In regard to age-related fertility decline in men, 57% believed that it simply did not happen. As to their desire to become parents, 58.3% stated that they planned to have children, and 50% said it was very important. The risk factors tied to infertility listed by the students were as follows: drug use (79.2%); aging (78.2%); smoking (69.2%); alcohol (66.5%); and sexually transmitted infections (43%).

**Conclusions:**

Argentinian university students wrongly see themselves as knowledgeable about fertility. Interventions are required to improve awareness over fertility among university students in Argentina.

## INTRODUCTION

Fertility awareness is a worldwide concern that has been studied in past years primarily in Europe and the United States (Pedro et al., 2018; Harper et al., 2017). Globally, women and men are delaying parenthood, with reasons ranging from women having broader access to further and higher education and jobs; couples choosing to focus on their careers, seek financial stability, or pursue other interests such as travelling; to difficulty finding a partner (Pedro et al., 2018; Lampic et al., 2006; Mills et al., 2011; Pierce & Mocanu, 2018). Over the past decades, the average age at which women become pregnant has consistently increased in the United States, Canada, and some European countries (Fritz & Jindal, 2018). Age-related declines in fertility and birth rates have become a major concern in areas such as health, economic development, and policy-making, particularly in developed nations (Pedro et al., 2018; Bunting et al., 2013; Alfaraj et al., 2019; Alaee et al., 2019).

The reasons for delaying parenthood have been traditionally associated with people attending university and wanting to pursue a career. Based on this, Lampic et al. (2006) decided to conduct a survey with Swedish university students to find out more about their intentions and attitudes towards parenthood and the knowledge they possessed about human reproduction. The authors found that male and female students had positive attitudes towards parenthood, and that they planned to start having children at the age of 35, which revealed a lack of awareness of the age-related decline in female fertility (Lampic et al., 2006). Several authors have looked into the matter, including Peterson et al. (2012) in the United States and Chan et al. (2015) in China. They found similar results, although the intentions of having children were significantly different among Chinese students, as they were less inclined to having offspring and less worried about infertility. These studies showed that most students wanted to have children in the future; however, they had significant knowledge gaps about fertility, especially in matters concerning female fertility and fertility decline. The authors also found that students tended to overestimate the age from which fertility declines and Assisted Reproductive Technology (ART) becomes a good option.

A study conducted in Canada looked into how effective online education was at increasing knowledge of fertility and assisted reproductive technology (Daniluk & Koert, 2015). The study enrolled 199 childless university students aged 18-35 years. They were asked to complete a survey that analyzed four beliefs through 22 questions asked before and immediately after the participants had taken an online training program on the subject. Post-program scores were significantly higher than pre-program scores, indicating that students had acquired knowledge from online education. However, six months later they repeated the survey and results showed that participants’ knowledge and beliefs regarding fertility and ART had regressed back to pre-training levels, particularly among males. Gender differences appear to be significant, since women scored higher in all areas. This might indicate that men downplay the importance of these matters, since reproduction, birth control and pregnancy tend to be traditionally regarded as more relevant for women. The decision to postpone reproduction increases the chances of remaining childless. Many couples that opted to have children at a later stage in their lives seek ART after unsuccessfully attempting to conceive (Fritz & Jindal, 2018). This was one of the reasons why Bunting & Boivin (2010) developed the FertiSTAT, an online tool that allows women to obtain personalized orientation about fertility, risk factors and recommendations on whether to look for medical help. There are other websites with similar purposes for men and women; one is “You Fertility,” developed by the Australian Government Department of Health and the Victorian Government Department of Health and Human Services as part of a national public educational program, the “Reproductive Life Plan” from Uppsala University.

Global concerns over increasing infertility rates and lack of knowledge about human reproduction have led to the inclusion of term “Fertility Awareness” (Zegers-Hochschild et al., 2017) in the glossary of the American Society for Reproductive Medicine (ASRM). The description of the term was based on the definitions proposed by the International Committee for Monitoring Assisted Reproductive Technology and the World Health Organization (Zegers-Hochschild et al., 2009), which described it as: “The understanding of reproduction, fecundity, fecundability, and related individual risk factors (e.g., advanced age, sexual health factors such as sexually transmitted infections (STIs), and lifestyle factors such as smoking, obesity) and non-individual risk factors (e.g., environmental and workplace factors); including the awareness of societal and cultural factors affecting options to meet reproductive family planning, as well as family building needs” (p. 400).

Many international studies have looked into the levels of fertility awareness and parenting intentions of university students (Lampic et al., 2006; Alfaraj et al., 2019; Peterson et al., 2012; Chan et al., 2015; Zhou et al., 2020), but none has described the situation in South America, with the exception of a Brazilian study that found that the fertility rate in Brazil is 1.73 vs. 2.49 at a global level (Nakagawa, 2018; Statista, 2020). This is a subject that deserves attention at a regional level. No studies on the subject have been carried out in Argentina, despite the existence of legislation regulating assisted reproductive technology. National legislation (Decreto 956/2013 Reglamentación de la Ley de Reproducción Médicamente Asistida, 2013; Ley 26.862 de Reproducción Médicamente Asistida, 2013) provides access to treatment to anyone aged 18+ years without an age limit, including same-sex couples, single women or men. Legislation covers access to ART, but it does not include information regarding fertility awareness; in fact, age is not contemplated as a barrier to having access to ART. According to the Argentine Society of Reproductive Medicine (SAMeR, 2017), 12,277 ART cycles have been performed and 7,819 led to embryo transfers. Forty-five percent of the pregnancies achieved involved women aged 35-40 years, and 30% involved women aged 40-45 years.

The latest census survey performed in the city of Buenos Aires (2013-2015) revealed a 30% increase in maternal age among the wealthier and more educated. In 2018, 685,394 babies were born, 84,768 from adolescent mothers (Ministerio de Salud Argentina, 2019). Unfortunately, Argentina lacks national statistics about the proportion of the population with infertility or the increase in the median age of first-time mothers, despite the enactment of the Comprehensive Sexual Education Law (Congreso de la Nación Argentina, 2006). From this data, it is possible to see a gap between the intention to give information and people acknowledging it. National Health authorities or medical associations have not organized campaigns to provide information and foster fertility awareness among youth. The Argentine Society of Reproductive Medicine (Pesce et al., 2017) focuses on medical or social reasons to postpone motherhood when it refers to fertility awareness, but the papers it publishes have the medical community as a target. The present study is the first to be conducted in Argentina to describe the level of fertility awareness, attitudes towards parenting, and knowledge about assisted reproductive technology among university students.

## MATERIALS AND METHODS

### Participants and procedures

This was a naturalistic, cross-sectional, quantitative study. Through a snowball design using social media (Instagram, Facebook, WhatsApp), university students were contacted to participate in the study in June 2019. Six hundred and eighty subjects completed an anonymous online, cross-sectional, self-report survey. Participants were mostly females (83.2%) and the mean age was 24.7 years (SD=5.6). Students were mainly from public universities (90%), studying diverse degrees and with domicile in the city of Buenos Aires (51%) or surrounding communities (49%). Most of individuals included in the study (93.9%) did not have children at the time they took the survey, and 56.8% were in a relationship.

### Measures

The authors translated the Swedish Fertility Awareness Questionnaire (Lampic et al., 2006) into Spanish and used it as a basis for data collection. Some items were adjusted or eliminated to fit the local cultural norm. First, a focus group interview was conducted with ten university students (eight women and two men) from different universities and career paths. Open questions were asked regarding what they knew about fertility, where they obtained information, and about legislation in Argentina. Afterwards, a pilot study was conducted, in which one question was adjusted for clarification.

The original version of the survey consisted of 56 questions. The final version of the questionnaire in Spanish had 37 questions covering the following domains:

Demographic data (7 items): Participants were required to state age, gender, city or area, university, degree, if they were in a relationship, and if they lived with a partner. 

Fertility awareness (12 items): Participants were asked to assess their knowledge about human reproduction and to answer where they obtained such information. The questions assessed their knowledge of female and male fertility at different ages, chances of achieving pregnancy, difficulties and associated risk factors.

Attitude towards parenthood (11 items): Participants were asked if they had children, if they would like to have them, how many and at what age. They were also asked if they had ever had an abortion, if having children was important to them, factors that might interfere with the decision to have children at ages of interest, and what they would do if they had fertility problems.

ART (7 items): Participants were asked to rate their knowledge about ART, its effectiveness, and the legal regulation regarding ART in Argentina.

### Data Analysis

SPSS v.21 was used in statistical analysis. Frequency analysis was used along with the T-test and the Mann-Whitney U-test. There were no significant differences between genders; therefore, only global results were presented. 

## RESULTS

The results were organized in three main areas according to the specific aims of the study: fertility awareness (level of knowledge about human reproduction and where participants obtained information on the subject; knowledge about risk factors associated with fertility); attitude towards parenting; and knowledge about ART. The results include the answers of the entire study population, as statistical tests failed to find significant differences in the answers provided by females and males to key questions. Age groups were compared with the same outcome.

### Fertility Awareness

Overall, 68.6% of the students believed they had high levels of knowledge about human reproduction (Table 1), and mentioned that most of their knowledge was acquired in social media or at school (Table 1).

**Table 1:** Fertility awareness

	%	n=669
Level of knowledge about human reproduction		
High	68.6%	459
Medium	29.6%	198
Low	1.8%	12
Source of knowledge		
Media	46.6%	312
School	24.2%	161
Family	11.1%	64
Friends	7.7%	51
Physician	5.9%	39
University	4.1%	27
Female fertility (cycle days)		
12-16	21.8%	146
Rest of days*	78.2%	523
Chances of pregnancy during ovulation		
0-20%	0.4%	3
20-40%	2.7%	18
40-60%	13.3%	89
60-80%	29.3%	196
80-100%	54.3%	363
Decrease in female fertility (years)**		
25-30	4.6%	31
35-40***	26%	174
40-45	33.2%	33.2
45-50	36%	241
Decrease in male fertility (years)		
35-45	1.2%	8
45-55	6.4%	43
55-65	13.9%	93
65-75	20.9%	140
Never	57.5%	385
Couples with difficulties achieving pregnancy (%)		
0-20%	10%	67
20-40%	33.8%	226
40-60%	45.6%	305
60-80%	10%	67
80-100%	0.6%	4

Notes:

* possible answers were divided ‘1-4; 4-8; 8-12; 12-16; 16-20; 20-24; 24-28’ and were counted as ‘rest of days’.

** n=668 (Only one person answered 20-35, and this data point was eliminated from the total.)

*** no one selected the 30-35 category

In terms of fertility, 78,2% considered that women were able to get pregnant at any moment of the cycle, and only 21.8% of the students indicated that it occurred from days 12-16 of the cycle. Also, 36.2% believed that fertility in women decreased between the ages of 45-50 years; 33.2% between the ages of 40-45; and 25.9% between the ages of 35-40 years. More than half of the students (54.3%) thought that the chances of getting pregnant during the ovulation period were around 80-100%. In relation to male age-related fertility decline, 57.5% thought it never decreased.

Finally, a great number of students (45.6%) answered that 40-60% of couples might experience difficulties achieving pregnancy, while 33.8% indicated that this might be an issue for 20-40% of the couples. 

**Figure 1:**
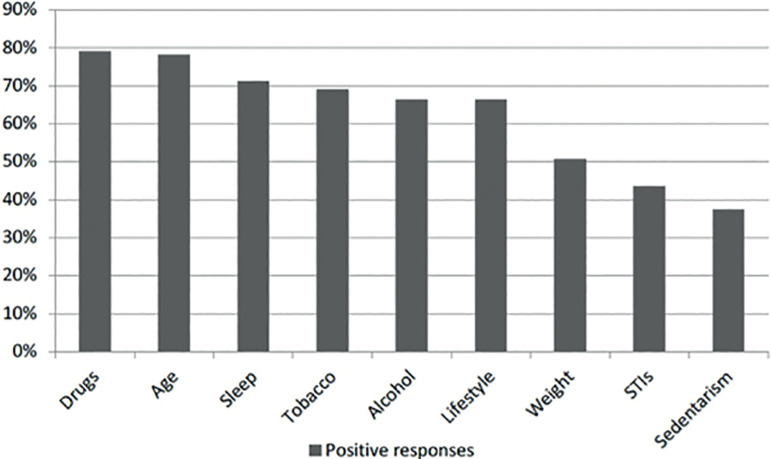
Risk factors associated with fertility.

In terms of fertility risk factors, the students listed drugs, age, tobacco and alcohol, and STIs, among others (Figure 1). Eating meat (4.3%) and being in contact with nature were presented as control options (1.8%) and, for that reason, were not included in Figure 1.

### Attitude towards parenting

Regarding parenting intentions, 60.2% of the childless participants stated they planned to become parents in the future, and 59.8% said that having children was very important for them. Typically, they wanted to have two (61.4%) or three (24%) children, and planned to have the first child before the age of 30 (41.9%); between the ages of 30-35 (48.6%); or between the ages of 35-40 (8.2%). When asked about the age at which they wanted to have their last child, they indicated primarily that they wish to have it before the age of 35 (45%) or 40 (43.2%) years, and 6.7% mentioned that they expected to have their last child over the age of 40. In general terms, they acknowledged three main threats to fulfilling their plans: financial problems (58.4%), achieving professional goals (55.1%), and not finding the right partner (43.6%). Fertility complications were not perceived as a possibility.

By contrast, 39.8% said that they did not plan to have children, for the following reasons (n=104): financial issues (33.7%), professional goals (21.2%), and not being able to fulfill the responsibility tied to being a parent (22.1%). 

### Knowledge about ART

Regarding fertility treatments, 26.8% of the students said they knew what ART was with some confidence, and 39.2% said that there was a chance of less than 40% that a single IVF cycle might be successful (Table 2). The vast majority (90%) indicated that they knew what egg freezing means. Finally, 47.4% were unaware that there was legislation regulating ART in Argentina.

**Table 2:** Knowledge about ART

	%	n=669
Knowledge about ART		
Yes	26.8%	279
No	28.1%	188
Some	45.1%	302
Causes of infertility		
Mixed	61.1%	409
Female	12.1%	81
Male	1.9%	13
Does not know	24.8%	166
Some	45.1%	302
Chances of having a live birth with one IVF cycle		
0-20%	11.2%	75
20-40%	28%	187
40-60%	38.9%	260
60-80%	19%	127
80-100%	3%	20
Knowledge about egg freezing		
Yes	90%	602
Yes	10%	67
Knowledge about ART legislation		
Yes	52.6%	352
No	47.4%	317

## DISCUSSION

The university students enrolled in this study saw themselves as knowledgeable about fertility. However, many believed that women could get pregnant at any moment of the cycle, made wrong assumptions about fertility decline in women, ignored what happened to men, and overestimated ART success rates. Our results were consistent with the results published in studies conducted with university students around the world (Pedro et al., 2018; Lampic et al., 2006; Peterson et al., 2012). The discrepancy between this study and others carried out in the United States and Europe is that almost half of the students did not intend to have children, which is surprisingly similar to the proportions reported for Chinese students (Chan et al., 2015). This might be explained by the current financial crisis in Argentina, as students reported economic hardship as the main reason not to have children or a potential obstacle to having children (Adsera & Menendez, 2011). Another explanation for this difference might be related to the years that separate studies performed on this subject, the first of which conducted in Sweden 14 years ago; the hypothesis that arises is that this gap may not be that wide today. An interesting finding is that students thought the chances of getting pregnant every cycle were very high, although almost half of them thought 40-60% of the population had difficulties achieving pregnancy. They claimed to have acquired knowledge about human reproduction mainly from social media or school, with a surprisingly small subset citing their physicians as the source of such information. This brings up the question of what the role of physicians is and, moreover, how much they know about it, as a study reported that residents overestimated the age at which female fertility declines and ART is successful (Yu et al., 2016).

Another surprising result is that half of the students did not acknowledge weight or STIs as risk factors, as both have been connected to decreased fertility and poorer health in general. Interestingly, they recognized other risk factors: substance abuse, tobacco, lifestyle and age, although the age they cited was incorrect, as they might potentially have associated it to menopause. Most of the students did not know what ART was. In an apparent contradiction, almost everyone said that they knew what egg freezing was. A limitation of the study is that follow-up questions regarding egg freezing and how successful they think it is were not included. Another possible limitation is that the students were from Buenos Aires, the city with most resources and access to education in Argentina.

In general terms, our study found that the included students lacked information about human reproduction, although they thought they had good knowledge about it, a phenomenon that requires additional analysis.

We believe this difference might be related to a cognitive avoidance mechanism, which might include distraction and thought suppression as strategies to avoid thoughts about undesired situations (Sagui-Henson, 2017). In this sense, students tended to avoid thinking about possible risk factors or future complications when attempting pregnancy. Additionally, attention at a global level has been given mostly to contraception, which might lead people to assume that it is easy to get pregnant. Sexual Education Law (Congreso de la Nación Argentina, 2006) in Argentina includes information about contraception and some risk factors such as STIs and substance abuse, although it does link them to how they may compromise fertility in the future or include age as a risk factor.

Nevertheless, what we do know is that in order to be able to make informed decisions, people need accurate information presented in a way they can understand it. Different resources were created to improve fertility awareness as previously mentioned (Bunting & Boivin, 2010; Your Fertility, 2020; Uppsala University, 2020; Nakagawa, 2018), but some had lackluster results (Daniluk & Koert, 2015). Locally, a step forward might include the adaptation of these resources to the Argentinian context, along with tailored interventions designed to reach more people, including the non-academic population and men.

This study showed that curricular changes are needed to improve fertility awareness among university students in Argentina. It also shed light on the complexity of the subject and its multiple causal links. Healthy lifestyle habits should be taught in schools and universities as part of a wider information program. The power of social media platforms should be harnessed to convey accurate information on human reproduction and policies are required to extend sexual and health education in universities and to include this subject in the public health agenda and discussions about reproductive rights.

## References

[r1] Adsera A, Menendez A (2011). Fertility changes in Latin America in periods of economic uncertainty. Popul Stud (Camb).

[r2] Alaee S, Yousefian E, Talaiekhozani A, Reza Ziaee G, Homayoon H (2019). Infertility Knowledge, Attitudes, and Beliefs among Iranian College Students. J Environ Treat Tech.

[r3] Alfaraj S, Aleraij S, Morad S, Alomar N, Rajih HA, Alhussain H, Abushrai F, Thubaiti AA (2019). Fertility awareness, intentions concerning childbearing, and attitudes toward parenthood among female health professions students in Saudi Arabia. Int J Health Sci (Qassim).

[r4] Argentina (2013). Decreto 956/2013. Reglamentación de la Ley de Reproducción Médicamente Asistida.

[r5] Argentina (2006). Ley 26.150. Programa Nacional de Educación Sexual Integral.

[r6] Argentina (2013). Ley 26.862. Reproducción Médicamente Asistida.

[r7] Argentina, Ministerio de Salud, Dirección de Estadísticas e Información en Salud (2019). Estadísticas vitales: información básica, 2018.

[r8] Bunting L, Boivin J (2010). Development and preliminary validation of the fertility status awareness tool: FertiSTAT. Hum Reprod.

[r9] Bunting L, Tsibulsky I, Boivin J (2013). Fertility knowledge and beliefs about fertility treatment: findings from the International Fertility Decision-making Study. Hum Reprod.

[r10] Chan CH, Chan TH, Peterson BD, Lampic C, Tam MY (2015). Intentions and attitudes towards parenthood and fertility awareness among Chinese university students in Hong Kong: a comparison with Western samples. Hum Reprod.

[r11] Daniluk JC, Koert E (2015). Fertility awareness online: the efficacy of a fertility education website in increasing knowledge and changing fertility beliefs. Hum Reprod.

[r12] Fritz R, Jindal S (2018). Reproductive aging and elective fertility preservation. J Ovarian Res.

[r13] Harper J, Boivin J, O’Neill HC, Brian K, Dhingra J, Dugdale G, Edwards G, Emmerson L, Grace B, Hadley A, Hamzic L, Heathcote J, Hepburn J, Hoggart L, Kisby F, Mann S, Norcross S, Regan L, Seenan S, Stephenson J (2017). The need to improve fertility awareness. Reprod Biomed Soc Online.

[r14] Lampic C, Svanberg AS, Karlström P, Tydén T (2006). Fertility awareness, intentions concerning childbearing, and attitudes towards parenthood among female and male academics. Hum Reprod.

[r15] Mills M, Rindfuss RR, McDonald P, te Velde E, ESHRE Reproduction and Society Task Force (2011). Why do people postpone parenthood? Reasons and social policy incentives. Hum Reprod Update.

[r16] Nakagawa HM (2018). Fertility awareness campaign. JBRA Assist Reprod..

[r17] Pedro J, Brandão T, Schmidt L, Costa ME, Martins MV (2018). What do people know about fertility? A systematic review on fertility awareness and its associated factors. Ups J Med Sci..

[r18] Pesce R, Marconi M, Vélez C, Marconi G, Glukovsky D, Baronio M, Coscia A (2017). Preservación de la fertilidad. Reproducción.

[r19] Peterson BD, Pirritano M, Tucker L, Lampic C (2012). Fertility awareness and parenting attitudes among American male and female undergraduate university students. Hum Reprod.

[r20] Pierce N, Mocanu E (2018). Female age and assisted reproductive technology. Glob Reprod Health.

[r21] Sagui-Henson SJ, Zeigler-Hill V, Shackelford T (2017). Cognitive Avoidance. Encyclopedia of Personality and Individual Differences.

[r22] SAMeR - Sociedad Argentina de Medicina Reproductiva (2017). Registro Argentino de Fertilización Asistida - RAFA. Resultados 2017.

[r23] Statista (2020). Global fertility rate from 2008 to 2018 [Internet].

[r24] Uppsala University (2020). Your reproductive life plan [Internet].

[r25] Your Fertility (2020). Understanding how to improve your chance of having a baby [Internet].

[r26] Yu L, Peterson B, Inhorn MC, Boehm JK, Patrizio P (2016). Knowledge, attitudes, and intentions toward fertility awareness and oocyte cryopreservation among obstetrics and gynecology resident physicians. Hum Reprod.

[r27] Zegers-Hochschild F, Adamson GD, de Mouzon J, Ishihara O, Mansour R, Nygren K, Sullivan E, van der Poel S, International Committee for Monitoring Assisted Reproductive Technology, World Health Organization (2009). The International Committee for Monitoring Assisted Reproductive Technology (ICMART) and the World Health Organization (WHO) Revised Glossary on ART Terminology, 2009. Hum Reprod.

[r28] Zegers-Hochschild F, Adamson GD, Dyer S, Racowsky C, de Mouzon J, Sokol R, Rienzi L, Sunde A, Schmidt L, Cooke ID, Simpson JL, van der Poel S (2017). The International Glossary on Infertility and Fertility Care, 2017. Fertil Steril.

[r29] Zhou Y, Luo Y, Wang T, Cui Y, Chen M, Fu J (2020). College students responding to the Chinese version of Cardiff fertility knowledge scale show deficiencies in their awareness: a cross-sectional survey in Hunan, China. BMC Public Health.

